# Dual Targeted Mitochondrial Proteins Are Characterized by Lower MTS Parameters and Total Net Charge

**DOI:** 10.1371/journal.pone.0002161

**Published:** 2008-05-14

**Authors:** Maya Dinur-Mills, Merav Tal, Ophry Pines

**Affiliations:** Department of Molecular Biology, Hebrew University Medical School, Jerusalem, Israel; Wellcome Trust Centre for Human Genetics, United Kingdom

## Abstract

**Background:**

In eukaryotic cells, identical proteins can be located in different subcellular compartments (termed dual-targeted proteins).

**Methodology/Principal Findings:**

We divided a reference set of mitochondrial proteins (published single gene studies) into two groups: i) Dual targeted mitochondrial proteins and ii) Exclusive mitochondrial proteins. Mitochondrial proteins were considered dual-targeted if they were also found or predicted to be localized to the cytosol, the nucleus, the endoplasmic reticulum (ER) or the peroxisome. We found that dual localized mitochondrial proteins have i) A weaker mitochondrial targeting sequence (MitoProtII score, hydrophobic moment and number of basic residues) and ii) a lower whole-protein net charge, when compared to exclusive mitochondrial proteins. We have also generated an annotation list of dual-targeted proteins within the predicted yeast mitochondrial proteome. This considerably large group of dual-localized proteins comprises approximately one quarter of the predicted mitochondrial proteome. We supported this prediction by experimental verification of a subgroup of the predicted dual targeted proteins.

**Conclusions/Significance:**

Taken together, these results establish dual targeting as a widely abundant phenomenon that should affect our concepts of gene expression and protein function. Possible relationships between the MTS/mature sequence traits and protein dual targeting are discussed.

## Introduction

In certain cases, identical or almost identical proteins can be found in more than one compartment, giving rise to isoprotein distribution [1]–[3]. A number of mechanisms can generate dual distribution between subcellular compartments. These mechanisms can be divided into two main groups based on the number of translation products that are generated. Isoprotein distribution due to two translation products can be achieved by several routes that are based on two genes, two mRNAs from a single gene or two translation initiations from a single mRNA. In all these cases the two isoproteins differ by the presence or absence of a targeting signal (reviewed in [1], [3]). Alternative situations involve the creation of a single translation product that harbors two targeting signals or an ambiguous signal that can target the protein to two organelles (reviewed in [2], [3]). There are also situations which involve changes in the accessibility of a signal, inefficient translocation or retrograde translocation driven by protein folding (reviewed in [2]).

Basic bioinformatic approaches designed to predict protein localization are based on local sequence pattern at the presumed location of targeting signals, mainly within the N or C-terminus. The existence of unconventional targeting signals in particular internal sequences has been known for some time [4], but an accurate identification of such signals in protein sequences is still not possible. Therefore proteins containing these atypical signals will most probably escape prediction using automated methods. A more direct approach to obtain the proteome of a subcellular compartment includes purification of a compartment and determination of protein content by mass spectrometry. While such an approach has been implemented successfully on mitochondria [5], [6] it is inapplicable for determining the endomembrane, cytosolic or nuclear proteomes. Other genetic approaches utilize systemic tagging of yeast genes and determining the localization of the fusion proteins by fluorescent microscopy. Tagging has been done by fusion of the green fluorescent protein (GFP) to the C-terminus [7]; by random transposon-mediated mutagenesis or by cloning of PCR-amplified open reading frames (ORFs) into an overexpression-tagging vector [8]. These approaches require that protein expression levels are sufficiently high to allow visualization and that the tags do not interfere with subcellular targeting. Furthermore overexpression as well as insertion of a tag can alter correct protein localization [6].

Here we have divided a reference set of previously reported mitochondrial proteins into two groups; predicted to be dual localized or exclusively mitochondrial, based on published experimental genomic screens and bioinformatic predictions of targeting signals. We find that dual targeted proteins constitute a separate subgroup within the mitochondrial proteome that is enriched for specific traits of their targeting signals or mature polypeptide sequences. In addition, we compiled a dataset of predicted dual targeted mitochondrial proteins which comprises approximately one quarter of the predicted yeast mitochondrial proteome.

## Materials and Methods

### Experimental procedures

#### Compilation of a dataset of mitochondrial proteins

The list of yeast ORFs and protein annotations are based on information in the *Saccharomyces* Genome Database (SGD; http://www.yeastgenome.org/).

Mitochondrial localization is determined by a reference set, based on single gene studies, available at the MitoP2 database (http://www.mitop.de). Evaluation of the mitochondrial proteome is based on the MitoP2 database with an SVM score >0.5; 1). Simple evaluation of mitochondrial localization is based on databases specified in Table 1. Proteins were designated as mitochondrial if they met at least two criteria and if at least one of these criteria belonged to the genome wide experimental screens (Mitochondrial proteomics; Subcellular localization screens). For statistical assessment of predicted groups we utilized the MitoP2 reference set. Specificity is defined as the proportion of proteins of a dataset which are part of the reference set, while sensitivity is the proportion of reference set proteins which is covered by the dataset.

**Table 1:** Criteria for the prediction of a dual-targeted mitochondrial proteome.

Protein localization	Database type	Methodology	Reference
1st location: Mitochondria Reference set	MitoP2	Single gene study	http://www.mitop.de
1st location: Mitochondria MitoP2	MitoP2 SVM (support vector machine)	MitoP2 SVM >0.5	http://www.mitop.de
1st location: Mitochondria Simple evaluation	Mitochondrial proteomics	Mitochondria purification +2D PAGE / Mass Spectrometry	Sickmann et al 2003
	Subcellular localization screens	Chromosomally GFP-tagged Proteins	Huh et al 2003
		Transposon-Insertion Phenotypes, Localization and Expression (TRIPLE)	Kumar et al 2002
	Null mutant phenotype	Pet mutants	Dimmer KS et al 2002
	In-Silico predictions	MTS prediction – MitoProtII >0.7	http://ihg.gsf.de/ihg/mitoprot.html
		Homology to the prokaryotic ortholog starts after 10–80 amino acids	blast
2^nd^ location	Subcellular localization screens	Chromosomally GFP-tagged Proteins	Huh et al 2003
		Transposon-Insertion Phenotypes, Localization and Expression (TRIPLE)	Kumar et al 2002
	In-Silico predictions	Peroxisome targeting sequence prediction – PTS1 predictor	http://mendel.imp.univie.ac.at/mendeljsp/sat/pts1/PTS1predictor.jsp
		ER signal peptide prediction - TargetP	http://www.cbs.dtu.dk/services/

#### Compilation of a dataset of dual-localized proteins

Proteins were first designated as mitochondrial as mentioned above and then their second location was determined if they met at least one of the criteria described at Table 1. For statistical assessment of predicted groups, as mentioned above, we generated a reference set of 29 dual-localized proteins by screening all published single gene studies.

#### Statistical analysis

Statistical analysis was performed using the SPSS Package (v.13, SPSS Inc., Chicago, IL) as implemented on a Windows XP platform. Analyzed parameters do not have a normal distribution. Therefore, a two tailed Mann Whitney U test for two independent samples was run to test the statistically significant differences between groups. Continuous parameters were categorized and Chi-square test was run to test differences in distribution.

#### Strains and plasmids

CW04 (*MAT* α, *leu2-3; ura 3-1; trp1-1; ade1-2; his3-11,15; can1-100*). BY4741 (*Mat a; his3-1; leu2-0; met15-0; ura3-0*). pWc (pYES/M15) was kindly provided by Picard [9], pFumα, pHxk1α and pKgd1α were described elsewhere [10]. BS-Su9w [10] was cut with KpnI and NotI and cloned into pYes2 to create pWm. pPrd1α, pHnt2α, pMge1α, pFmp40α, pGlo4α, pGpd2α, pLpd1α, pAep1α, pYgr031wα, Fmp36α, Mss116α, Acn9α, Mrpl11α and Gcv3α were created by amplifying the corresponding ORFs by PCR with the primers specified in Table S5 and using yeast genomic DNA as the template. The resulting products were cloned into pFumα using an Orientation Enrichment Reaction (OER) (Gene Bio Application Ltd., Kfar Hanagid, Israel). All plasmids described above were introduced into strain CW04.

#### Growth conditions

Strains were grown at 30°C or as indicated in synthetic depleted medium containing 0.67% (w/v) yeast nitrogen base without amino acids (Difco Laboratories), 2% glucose or galactose (w/v), CSM dropout mix (Qbiogene) supplemented with the appropriate amino acids (50 μg/ml). For agar plates, 2% agar was added. X-gal plates were prepared as above 2% galactose, 1% raffinose, 0.008% X-gal (dissolved in 100% *N*,*N*-dimethylformamide) and 1×BU salts (25 mM sodium phosphate buffer titrated to pH 7.0) were added after medium was autoclaved and cooled down.

#### β-Galactosidase α-complementation plate assay

Yeast cells were transformed with plasmids encoding various α fusion proteins and either ω_c_ or ω_m_. Colonies were plated on X-gal plates and incubated at 30°C for 72 h.

## Results

### Dual-localized mitochondrial proteins have a lower MitoProtII score

The MitoP2 database (http://www.mitop.de) [11], [12] offers a reference set of 535 mitochondrial proteins, annotated manually according to published experimental data. We used the following logic: We considered a protein as dual localized if it was implied to be in a second location according to genomic screens including GFP tagging, the TRIPLE database (Transposon-Insertion) or bioinformatic predictions of targeting signals. As shown in Table 1, 126 proteins are considered to be dual targeted and 409 exclusively mitochondrial.

The MitoProtII program (confusing similar name, but it is not the MitoP2 database above) produces a score that represents the probability that a protein is mitochondrial based on analysis of its N terminal sequence and characteristics of the whole protein (http://ihg.gsf.de/ihg/mitoprot.html; [13]). We analyzed the dual-localized and exclusive-mitochondrial groups, using this program. The dual-localized proteins have a statistically significant lower median score, 0.603 compared to 0.896 of the exclusive mitochondrial proteins (p-value <0.001 by Mann-Whitney; Table 2). This score is also lower than the median score for total mitochondrial proteins (0.868). A similar pattern is observed when examining the mean values of these groups (Table 2). Thus we conclude that dual-localized mitochondrial proteins have a lower MitoProtII score. One concern we had was that membrane proteins and intermembrane space proteins may cause a bias due to their unique traits of targeting and membrane insertion. Nevertheless, when we remove from the analysis membrane proteins (140 by GO annotation) or intermembrane space proteins (36 by GO annotation) or both simultaneously, we still find a statistically significant difference between MitoProtII scores of dual and exclusive mitochondrial proteins (not shown).

**Table 2:** Comparison of MitoProtII scores of dual localized versus exclusive mitochondrial proteins in the mitochondrial reference set.

Protein location	MitoProtII Score					
	N	Median	Mean	SD	p-value* (Mann-Whitney)	p-value* (χ2 score, df)
Mitochondrial proteins	535	0.868	0.660	0.37	-	-
Predicted exclusive mitochondrial proteins	409	0.896	0.698	0.36	**<0.001**	0.009 (21.9, 9)
Predicted dual localized mitochondrial proteins	126	0.603	0.538	0.40		

MitoProtII scores (which represent the probability that a protein is mitochondrial) were calculated for proteins of the mitochondrial reference set. MitoProtII median and mean values with their standard deviation of exclusive mitochondrial and dual localized proteins are shown. Significance of differences between the medians of exclusive mitochondrial and dual localized groups was determined by the Mann Whitney test (bold p-value). MitoProtII scores were categorized and Chi-square test was run to test differences in distribution (bottom right). χ2-p-value, is shown with the χ2 score and degrees of freedom (df) in brackets respectively. ^*^ Differences are considered significant if p-value <0.05.

**Figure 1:**
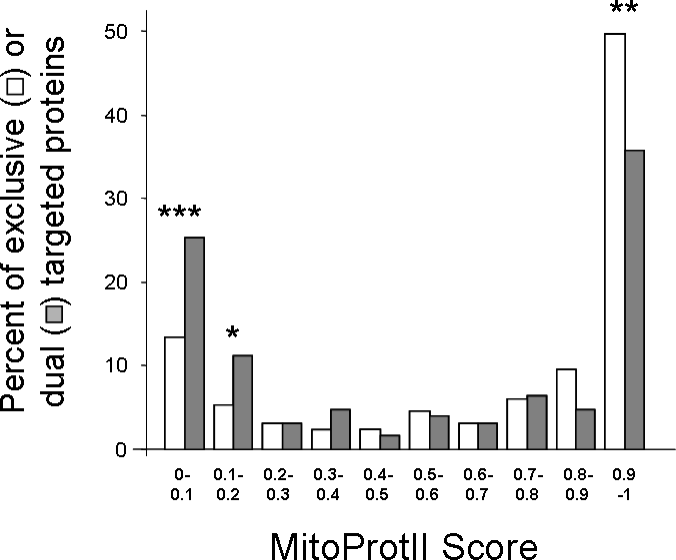
Dual localized proteins of the mitochondrial reference set are enriched for proteins with a low MitoProtII score. Distribution of MitoProtII scores in dual localized (grey) and exclusive mitochondrial (white) proteins were analyzed using χ^2^ test. Statistically significant differences in specific categories according to the χ^2^ test (df = 1) are marked with asterisks (* p-value <0.05; ** p-value <0.005; *** p-value <0.001). Mitochondrial localization was determined according to the Mitop2 reference set.

We divided the two groups of the reference set above (dual targeted and exclusive) into subgroups of MitoProtII score intervals and examined the difference in their distribution. (Fig. 1; Table 2, χ2 test results). Dual-localized proteins are enriched for proteins harboring a weak MitoProtII score (<0.2), whereas, exclusive mitochondrial proteins are enriched in proteins harboring a strong MitoProtII scores (>0.7) (Apparent in Fig 1). This difference in distribution is statistically significant according to χ2 test (p-value = 0.009, Table 2).

### Dual-localized mitochondrial proteins are likely to harbor a weak MTS

Import of many nuclear encoded mitochondrial proteins is mediated by a mitochondrial targeting sequence (MTS). This N terminal presequence is usually enriched in positive, hydrophobic, and hydroxylated amino acids and devoid of acidic amino acids. It is also characterized by a tendency to form an amphiphylic α-helix, which presents one positively charged surface and one hydrophobic surface [14]–[16].

We analyzed several parameters of the N terminal sequences: i) The hydrophobic moment (μHδ) which is used as a measure of the helical amphiphilicity or the asymmetry of the distribution of hydrophobic side chains [16], ii) The maximal hydrophobicity (Hmax) of the hydrophobic face of the helical structure [16] and iii) The number of positively charged residues within the N-terminus. Compared to exclusive-mitochondrial proteins, dual-localized proteins contain a statistically significantly lower median hydrophobic moment (5.905 versus 7.387, p-value <0.001) and a statistically significant lower number of positively charged amino acids (5.00 versus 6.00, p-value = 0.005) (Table 3, Mann-Whitney test). We also observed a lower median Hmax value (4.385 versus 4.71), however this difference is not statistically significant (p-value = 0.06). Again, similar tendencies are observed when examining differences in the mean values of these groups (Table 3).

**Table 3:** Comparison of MTS parameters of dual localized versus exclusive mitochondrial proteins in the mitochondrial reference set.

Parameter	Protein location	N	Median	Mean	SD	p-value* (Mann-Whitney)	p-value* (χ2 score, df)
μHδ	Mitochondrial proteins	535	7.064	6.856	2.578	-	-
	Predicted exclusive mitochondrial proteins	409	7.387	7.099	2.46	**<0.001**	<0.0026 (25.3, 9)
	Predicted dual localized mitochondrial proteins	126	5.905	6.064	2.79		
Hmax	Mitochondrial proteins	535	4.61	4.431	1.72	-	-
	Predicted exclusive mitochondrial proteins	409	4.71	4.521	1.62	**0.06**	0.11 (10.2, 6)
	Predicted dual localized mitochondrial proteins	126	4.385	4.136	1.98		
Number of positively charged residues in N-terminus	Mitochondrial proteins	535	6.00	5.94	4.189	-	-
	Predicted exclusive mitochondrial proteins	409	6.00	6.22	4.16	**0.005**	0.01 (27.5, 13)
	Predicted dual localized mitochondrial proteins	126	5.00	5.02	4.15		

The hydrophobic moment (μHδ), maxmimal hydrophobicity (Hmax), and the number of positive charged residues in the N-terminus are parameters used to evaluate the strength of mitochondrial targeting sequences (MTS, see text). Statistical analysis of differences between parameters of dual and exclusive mitochondrial proteins was carried out as in Table 2.

We divided the two groups of proteins (dual targeted and exclusive) into subgroups and revealed that dual localized proteins are enriched with proteins with low hydrophobic moment values (μHδ <6), and proteins with less than 3 positively charged residues in their N-terminus. These differences are reflected in the statistically significantly different distribution of these parameters (Table 3, χ2 test, p-value <0.0026 for μHδ; p-value <0.01 for the number of positively charged residues).

### Dual localized proteins are more negatively charged than exclusive mitochondrial proteins

Early reports described a positive net charge difference between the mature polypeptide sequences of mitochondrial proteins and their homologous cytosolic isoproteins. [17], [18]. Our analysis shows that dual localized proteins have a statistically significant lower median net charge compared to exclusive mitochondrial proteins (3.0 versus 7.0, p-value = 0.0024) (Table 4, Mann-Whitney test). When using the predicted mitochondrial proteome (see below) rather than the mitochondrial reference set, an even higher statistical significance is observed (p-value = 5×10^−9^),

**Table 4:** Comparison of the total net charge of dual localized versus exclusive mitochondrial proteins of the reference set.

Protein location	Total Net Charge					
	N	Median	Mean	SD	p-value* (Mann-Whitney)	p-value* (χ2 score, df)
Mitochondrial proteins	535	6.00	6.80	11.82	-	-
Predicted exclusive mitochondrial proteins	409	7.00	7.29	10.51	**0.0024**	0.032 (10.6, 4)
Predicted dual localized mitochondrial proteins	126	3.00	5.21	15.26		

Dual targeted mitochondrial proteins have a lower total protein net charge than exclusive mitochondrial proteins. Statistical analysis of differences between parameters of dual and exclusive mitochondrial proteins was carried out as in Table 2.^*^ Differences are considered significant if p-value <0.05.

**Figure 2:**
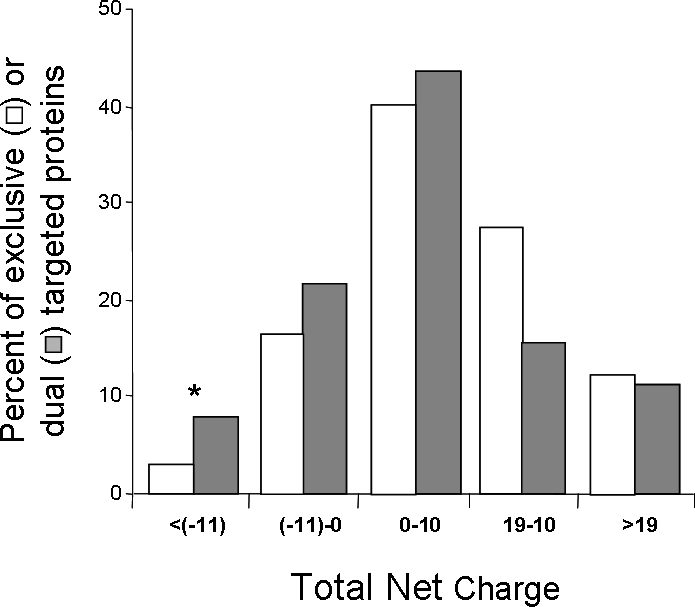
Dual localized proteins of the mitochondrial reference set are enriched for proteins with a low total net charge. Total Differences in the distribution of total net charge in dual localized (grey) and exclusively mitochondrial (white) proteins were analyzed using χ^2^ test (p-value <0.001). Statistically significant differences in specific categories according to the χ^2^ test (df = 1) are marked with asterisks (* p-value <0.05). Mitochondrial localization was determined according to the Mitop2 reference set.

Examination of subgroups of the dual targeted and exclusive mitochondrial proteins reveals that dual localized proteins are enriched for proteins with a low and negative net charge (Table 4, χ2 test, p-value = 0.032, Fig 2). These results suggest that dual targeting of mitochondrial proteins is mediated not only by an N-terminal targeting signal but also by properties of the entire protein such as its net charge.

### Dual targeted mitochondrial proteins with a strong MTS are more likely to have two translation products

Dual targeted proteins appear to have a higher probability (than exclusive mitochondrial proteins) for a weak MTS. Nevertheless, there is still a significant portion of mitochondrial dual targeted proteins that have a strong MTS. We assume that these proteins are more likely to have two translation products while exclusive mitochondrial proteins are more likely to have a single translation product. The rationale is that for dual-localized with two translation products, one longer product has an MTS while the other lacks an MTS. The longer product in this case can harbor a strong MTS since it will end up in mitochondria while the shorter product lacking the signal will end up in the cytosol. In other words dual targeting in these cases is determined by the presence or absence of an MTS rather than by its weakness.

Known dual targeted proteins with two translation products harbor a second, AUG-methionine codon, (potential translation initiation codon) in the interval of amino acids 8–60. Thus, a significant difference in the presence of a second methionine should be evident only when a strong MTS is present at the amino terminus.

We divided the mitochondrial reference set proteins according to their MitoProtII scores. The number of proteins containing a strong score (MitoProtII score 0.7 – 1), and a second methionine between residues 8–60 is 1.32 times higher for dual localized than for exclusive mitochondrial proteins. On the other hand, proteins without a second methionine between residues 8–60 are 1.28 times less dual localized than exclusive mitochondrial proteins. This difference is statistically significant (p-value = 0.047, one tailed χ2 test).

As described in detail in the next sections we have compiled a predicted dataset of dual targeted proteins within the yeast mitochondrial proteome. It is important to point out that our findings regarding the second methionine are true and more statistically significant for proteins from the predicted dataset of dual targeted proteins. For such proteins containing a strong score (MitoProtII score 0.7 – 1) there is even a more statistically significant difference in the presence of a second methionine between dual-localized and exclusive mitochondrial proteins (p-value = 0.006, one tailed χ2 test).There were no statistically significant differences in the presence of a second methionine in proteins with low or intermediate strength MTSs (MitoProtII score <0.2; 0.2–0.7).

### Compilation of a reference set of yeast dual localized mitochondrial proteins (Dual-Ref-Set)

We generated a reference set of 29 dual localized mitochondrial proteins whose localization was determined by screening all published single gene studies. For clarity we term this set, “Dual-Ref-Set”. Table S4 in the supplementary material offers an annotated list of this Dual-Ref-Set and additional information regarding the targeting mechanism, known targeting motifs and characterization of the predicted mitochondrial targeting signal (MTS). Worth mentioning, is that the Dual-Ref-Set proteins have similar traits to those discussed for predicted dual-targeted proteins (low MitoProtII score, MTS parameters and net charge) with similar mean and median values.

Of the 29 proteins comprising the Dual-Ref-Set, Twelve proteins are proposed to have two translation products initiated from a downstream AUG. The majority of 9 of these 12 proteins contain a strong predicted MTS characterized by a MitoProtII score stronger than 0.5. In only three cases is the MTS very weak and characterized by a MitoProtII score of less than 0.1.

Nine proteins of the 29 Dual-Ref-Set are proposed to have a single translation product. The majority (six) of these nine proteins with single translation products, have a very weak MitoProtII score of less than 0.1 and only three have a strong score. The trend of all these observations agreeably follows our analysis regarding the second methionine of dual targeted proteins in the previous section.

### Dual-localized proteins encompass a quarter of the mitochondrial proteome

We sought to compile a predicted dataset of dual targeted proteins within the yeast mitochondrial proteome. To do so we first used predictions of the mitochondrial proteome and then as before asked which of these proteins are also localized to other subcellular compartments including the cytosol, nucleus, endoplasmic reticulum (ER), and peroxisome. The mitochondrial proteome was predicted using the MitoP2 database which integrates information on mitochondrial proteins, their molecular functions and associated diseases (http://www.mitop.de) [11], [12]. This reference set is used for calculations of specificity and sensitivity of mitochondrial proteome predictions. The MitoP2 database utilizes a support vector machine (SVM) trained to classify protein localization. Using a default of SVM score >1, we estimated that the mitochondrial proteome consists of 540 proteins of which 431 are found in the mitochondrial reference set resulting in a specificity of 79.8% and a sensitivity of 80.6%. In order to achieve reasonable values of specificity and sensitivity for predicted dual-targeted proteins, we also used an SVM score of 0.5 which yields a list of 692 proteins (Table 5). Of this group 470 proteins are found in the mitochondrial reference set resulting in a specificity of 67.9% and a sensitivity of 87.8%.

To identify putative mitochondrial proteins that may also be located in a second subcellular location, we considered proteins as dual localized if they were found in a second location according genomic screens including GFP tagging, the TRIPLE database or bioinformatic predictions of targeting signals to the peroxisome and ER (Table 1).

**Table 5:** Prediction of dual localized mitochondrial proteins.

Mitochondrial proteome	Number of predicted dual localized proteins	% of predicted mitochondrial proteome	Number of predicted proteins in the dual-localized reference set	Specificity (%)^a^	Sensitivity (%)^b^
MitoP2 SVM >1	106	19.8	14	13.2	48.3
MitoP2 SVM >0.5	188	27.2	18	9.6	64.3
Simple evaluation	181	26.9	20	10.9	68.9

Prediction of dual localized mitochondrial proteins are shown using different predicted mitochondrial proteomes.

a Specificity is defined as the proportion of proteins of a dataset which are part of the dual-localized reference set

b sensitivity is defined as the proportion of the dual-localized reference set proteins which is covered by the dataset

Using the MitoP2 database (SVM>0.5) to determine mitochondrial localization, we estimate that there are 188 (27.2%) dual localized mitochondrial proteins of which 18 are found in the Dual-Ref-Set resulting in a specificity of 9.6% and a sensitivity of 64.3%. In addition to the MitoP2 database we compiled a simplistic evaluation of the mitochondrial proteome (Table 1). Using this simple evaluation, slightly higher results are obtained in which case we get an estimate of 181 dual-localized proteins of which 20 are found in the Dual-Ref-Set resulting in a specificity of 10.9% and a sensitivity of 68.9% (Table 5). Taken together, it appears that dual-targeted proteins constitute a substantial group comprising a quarter of the mitochondrial proteome.

It is important to state that dual-localized and exclusive mitochondrial proteins based on MitoP2 (SVM>0.5) or our simplistic evaluation for mitochondrial localization show the same traits that were found for the two groups based on the mitochondrial reference set: Dual-localized mitochondrial proteins have statistically significant lower MitoProtII scores compared to exclusive mitochondrial proteins, lower MTS parameters and a lower net charge. Accordingly there is a significant enrichment of proteins harboring a lower MitoProtII score, lower MTS parameters and lower net charge (Tables S1, S2, S3).

### Experimental verification of dual/exclusive subcellular targeting by α-complementation

To test predicted dual localized proteins and in particular those with strong MTSs (referred to in the previous sections), we experimentally assessed a subgroup of 14 soluble proteins. These proteins were suggested by our analysis, to be either dual - localized to the mitochondria and cytosol (9 genes, Table S5A) or exclusively mitochondrial (5 genes, Table S5B). Proteins chosen were predicted to have a strong MTS and to be soluble which would ensure easier analysis. Prediction of an MTS was determined using the MitoProtII database with a cutoff score of 0.7 and whose prokaryotic orthologs are shorter by 10–80 amino acids. The rationale for the latter criterion is that during evolution mitochondrial proteins from prokaryotic origin needed to evolve a targeting signal in order to enter the mitochondria and this signal is often located as an addition at the N-terminus, preceding the structural protein. Membrane bound proteins were excluded due to the complexity of their analysis.

Analysis of these genes involved a compartment specific α-complementation assay which has been demonstrated as a simple and sensitive method for probing the dual protein localization within yeast cells [10]. The basis of this approach is the requirement for localization of complementing β-galactosidase fragments (α – 77 amino acids; ω – 993 amino acids) within the same compartment to achieve enzymatic activity. The α fragment was attached to the C terminus of each of the 9 proteins. The fusions were cloned into a yeast expression plasmid under the regulation of an inducible *GAL10* promoter. The various α fusions were co-expressed in yeast cells with a cytosolic (ω_c_) or mitochondrial (ω_m_) ω fragment. Upon growth on plates containing X-gal, the cells gave a color phenotype presumably representing the localization of the α fusion protein.

**Figure 3:**
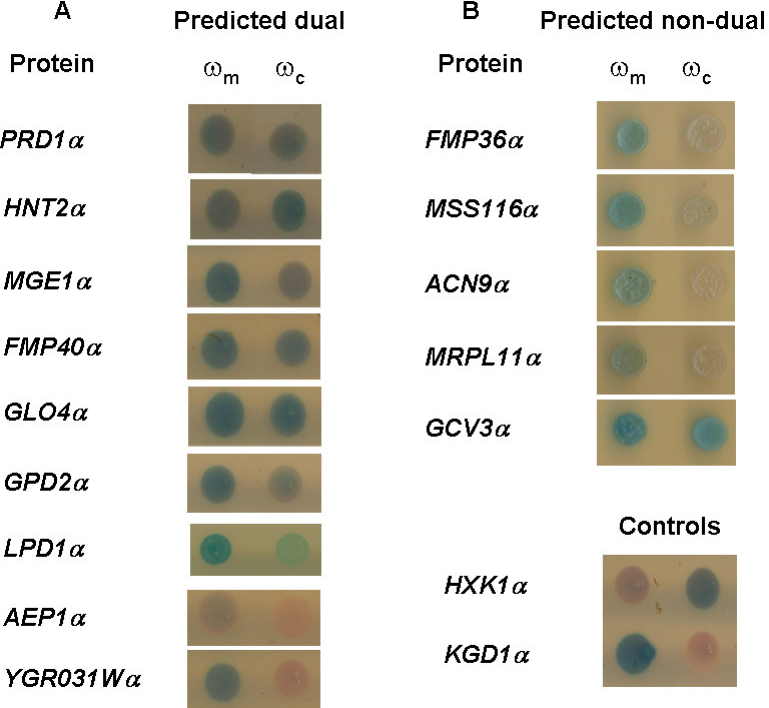
α-complementation assay for mitochondrial and cytosolic location of predicted dual (A) and nondual (B) localized proteins. Yeast expressing the indicated α fusion proteins and the ωc or ωm fragments were tested for color production on galactose medium, X-gal agar plates.

Seven out of the nine proteins predicted to be dual-localized to mitochondria and the cytosol (Fig. 3A) exhibited a color phenotype corresponding to the expected dual localization, while two appeared to be exclusively mitochondrial. In comparison only one protein (GCV3) predicted to have an exclusive mitochondrial location exhibited dual localization phenotype while four out of these five proteins exhibited as predicted an exclusive mitochondrial phenotype (Fig. 3B). These results support our contention of abundant dual targeting of mitochondrial proteins in yeast.

It was pleasing to find that the three proteins (two of the dual-targeted and one of the exclusive mitochondrial) that according to α-complementation did not fit our initial prediction of subcellular location, did however display an expected net charge in agreement with the α-complementation. AEP1 and YGR0131W have a net charge of 18 and 12 respectively (Table S5A) which suits exclusive mitochondrial proteins while GCV3 has a net charge of −11 (Table S5B) which suits dual targeted proteins.

## Discussion

This study shows that dual localized mitochondrial proteins are characterized by a lower probability of mitochondrial targeting (MitoProtII score) and weaker MTS parameters when compared to exclusive mitochondrial proteins. Actually there is a highly significant enrichment in proteins harboring a weak MTS within the predicted dual targeted proteins. Thus, dual-targeted mitochondrial proteins appear to constitute a subgroup of mitochondrial proteins with distinctive properties.

**Figure 4:**
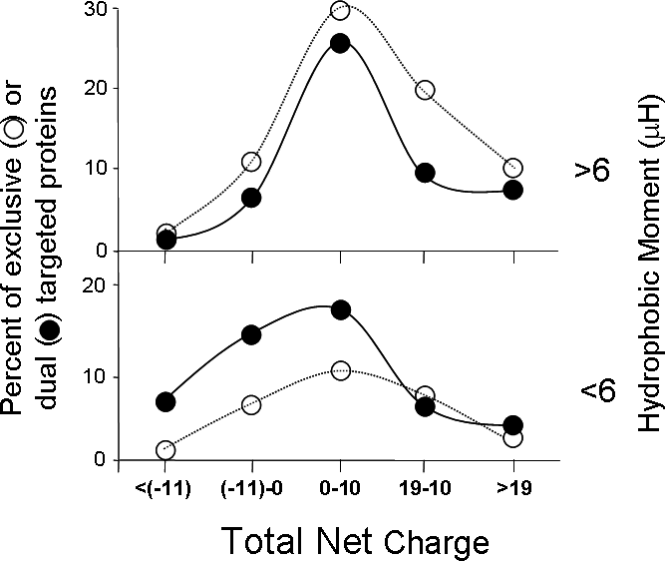
Proteins with a low net charge and low hydrophobic moment tend to be dual localized (more than exclusive mitochondrial proteins). Differences in the distribution of total net charge in dual localized (black circles) and exclusively mitochondrial (white circles) proteins with either high (>6, top panel) or low (<6, bottom panel) hydrophobic moment were analyzed using χ^2^ test. For proteins with a low hydrophobic moment (<6) there is a statistically significant difference between dual localized (black) and exclusively mitochondrial (white) proteins (p-value<0.02).

Although the strength of the MTS is a predominant feature in mitochondrial targeting, other factors also play a role in this process. The net charge of the whole protein is higher in mitochondrial proteins, even though the molecular understanding underlying this observation is lacking. It has been suggested that this may reflect an evolutionary selection on proteins to accommodate the mitochondrial matrix's higher pH [17], [18]. In this regard dual targeted proteins as one would expect have a lower net charge compared to exclusive mitochondrial proteins. Thus, not only the N-terminal targeting signal but also by the entire protein properties (such as its net charge), mediate dual targeting of mitochondrial proteins. Shown in Fig. 4 is a graphic representation that refers to total net charge, hydrophobic moment (as an indication of MTS strength) and the percent of proteins (dual or exclusive) at each value. This representation shows that proteins with a low net charge and weaker MTS tend to be dual localized (rather than exclusive mitochondrial proteins).

Dual-targeted proteins can not obviously be referred to as a homogenous group and in fact fall into two well separated groups according to the strength of their MTS; very high and very low MitoProtII scores. Since dual targeting is achieved by different mechanisms one would like to correlate these MTS-groups with specific mechanisms of subcellular distribution. These findings will certainly be the basis for future studies. Here, we asked whether proteins with two translation products (one containing and one lacking the MTS) are likely to have a strong MTS since it is the presence (or absence) of the signal that determines distribution. In fact we find a statistically significant enrichment in a second methionine between residues 8–60 in such dual-targeted (strong MTS containing) proteins. In contrast, in proteins harboring a weak or no classical MTS there is no such enrichment in a second methionine. One can also consider these notions by examining the Dual-Ref-Set (reference set of dual-localized mitochondrial proteins). Twelve proteins are proposed to have two translation products initiated from a downstream AUG. Nine of them contain a strong predicted MTS while only in three cases is the MTS very weak. In contrast, nine proteins are proposed to have a single translation product of which six have a very weak MitoProtII score and three have a strong score. In this regard a weak MTS might be involved in the mechanism of dual targeting by causing inefficient mitochondrial targeting as for example is the case in ADK1 [19]–[21]. Since the Dual-Ref-Set and in particular proteins whose mechanism of distribution has been studied is small (21 proteins) these differences are not statistically significant but are consistent with the notions of the MTS role in dual targeting and can certainly lead us to future avenues of investigation.

We have generated an annotation list of dual-targeted proteins within the predicted yeast mitochondrial proteome. The prediction is supported by the Dual-Ref-Set based on published single gene studies and by experimental verification of a subgroup of predicted dual targeted proteins. Strikingly, there is a considerably large group of dual targeted proteins which comprise approximately a quarter of the mitochondrial proteome. These results should change the way we refer to dual targeting, not merely as a rare event but as a widely abundant phenomenon affecting our concepts of gene expression and protein function.

The dual targeted mitochondrial proteome may even be larger than estimated above. Screens based on cell visualization (such as GFP tagging or the TRIPLE database) are the major source of information in the prediction of mitochondrial proteins' dual distribution. These screens tend to overlook “eclipsed distributed” proteins in which a large sub-population of a protein in one location obscures detection of a minute sub-population in a second location (24, 25). In this regard our examination of a subgroup of predicted exclusive mitochondrial proteins reveals that one out of five proteins is dual localized according to the α-complementation assay. Hence, due to a limitation in the sensitivity of the current screening methods, the dual targeted proteome might be even larger than currently predicted due to eclipsed distribution. This problem may be partially relieved by developing screens based on shorter tags and split reporter genes such as those developed for β-galactosidase [10] and GFP [22], [23].

## Supporting Information

Table S1 MitoProtII scores (which represent the probability that a protein is mitochondrial) were calculated for predicted mitochondrial proteins. MitoProtII median and mean values with their standard deviation of exclusive mitochondrial and dual localized proteins are shown. Significance of differences between the medians of exclusive mitochondrial and dual localized groups was determined by the Mann Whitney test (bold p-value). MitoProtII scores were categorized and Chi-square test was run to test differences in distribution (last column). χ2-p-value, is shown with the χ2 score and degrees of freedom (df) in brackets respectively. Significance of differences between the medians of mitochondrial (Third row) and non-mitochondrial proteins (bottom row) is shown on the right hand side of the bottom row. * Differences are considered significant if p-value <0.05.
(0.03 MB DOC)

Table S2 The hydrophobic moment (μHδ), maxmimal hydrophobicity (Hmax), and the number of positive charged residues in the N-terminus are parameters used to evaluate the strength of mitochondrial targeting sequences (MTS, see text). Statistical analysis of differences between parameters of dual and exclusive mitochondrial proteins was carried out as in Supplementary Table S1.
(0.05 MB DOC)

Table S3 Dual targeted mitochondrial proteins have a lower total protein net charge than exclusive mitochondrial proteins. Statistical analysis of differences between parameters of dual and exclusive mitochondrial proteins was carried out as in Supplementary Table S1.* Differences are considered significant if p-value <0.05.
(0.03 MB DOC)

Table S4 (0.25 MB DOC)

Table S5 (0.05 MB DOC)
