# Supplementary material for: Dual Targeted Mitochondrial Proteins Are Characterized by Lower MTS Parameters and Total Net Charge
Source: PLoS One. 2008 May 14;3(5):e2161. doi: 10.1371/journal.pone.0002161 (PMC2367453; doi:10.1371/journal.pone.0002161)
Supplement: Table S1 — MitoProtII scores (which represent the probability that a protein is mitochondrial) were calculated for predicted mitochondrial proteins. MitoProtII median and mean values with their standard deviation of exclusive mitochondrial and dual localized proteins are shown. Significance of differences between the medians of exclusive mitochondrial and dual localized groups was determined by the Mann Whitney test (bold p-value). MitoProtII scores were categorized and Chi-square test was run to test differences in distribution (last column). χ2-p-value, is shown with the χ2 score and degrees of freedom (df) in brackets respectively. Significance of differences between the medians of mitochondrial (Third row) and non-mitochondrial proteins (bottom row) is shown on the right hand side of the bottom row. * Differences are considered significant if p-value <0.05. (0.03 MB DOC) [file pone.0002161.s001.doc]

Table S1: MitoProtII scores of dual localized versus exclusive mitochondrial proteins in predicted mitochondrial proteins.

| Predicted protein location | MitoProtII Score | | | | | |
| --- | --- | --- | --- | --- | --- | --- |
|  | N | Median | Mean | SD | p-value* (Mann-Whitney) | p-value*  (χ2 score, df) |
| Exclusive mitochondrial proteins | 492 | 0.92 | 0.74 | 0.33 | **< 0.001** | < 0.001  (40.9, 9) |
| dual localized mitochondrial proteins | 181 | 0.74 | 0.563 | 0.39 |  |  |
| Mitochondrial proteins | 673 | 0.89 | 0.69 | 0.36 | < 0.001 | < 0.001  (370.3, 9) |
| Non-mitochondrial proteins | 6026 | 0.098 | 0.224 | 0.27 |  |  |

MitoProtII scores (which represent the probability that a protein is mitochondrial) were calculated for predicted mitochondrial proteins. MitoProtII median and mean values with their standard deviation of exclusive mitochondrial and dual localized proteins are shown. Significance of differences between the medians of exclusive mitochondrial and dual localized groups was determined by the Mann Whitney test (bold p-value). MitoProtII scores were categorized and Chi-square test was run to test differences in distribution (last column). χ2-p-value, is shown with the χ2 score and degrees of freedom (df) in brackets respectively. Significance of differences between the medians of mitochondrial (Third row) and non-mitochondrial proteins (bottom row) is shown on the right hand side of the bottom row. * Differences are considered significant if p-value < 0.05.
