# Supplementary material for: Dual Targeted Mitochondrial Proteins Are Characterized by Lower MTS Parameters and Total Net Charge
Source: PLoS One. 2008 May 14;3(5):e2161. doi: 10.1371/journal.pone.0002161 (PMC2367453; doi:10.1371/journal.pone.0002161)
Supplement: Table S2 — The hydrophobic moment (μHδ), maxmimal hydrophobicity (Hmax), and the number of positive charged residues in the N-terminus are parameters used to evaluate the strength of mitochondrial targeting sequences (MTS, see text). Statistical analysis of differences between parameters of dual and exclusive mitochondrial proteins was carried out as in Supplementary Table S1. (0.05 MB DOC) [file pone.0002161.s002.doc]

Table S2: Comparison of MTS parameters of dual localized versus exclusive mitochondrial proteins in predicted mitochondrial proteins.

| Parameter | Predicted protein location | N | Median | Mean | SD | p-value* (Mann-Whitney) | p-value*  (χ2 score, df) |
| --- | --- | --- | --- | --- | --- | --- | --- |
| μHδ | Exclusive mitochondrial proteins | 492 | 7.57 | 7.339 | 2.45 | **< 0.001** | < 0.001  (44.4, 9) |
|  | dual localized mitochondrial proteins | 181 | 6.09 | 6.179 | 2.70 |  |  |
|  | Mitochondrial proteins | 673 | 7.29 | 7.027 | 2.57 | < 0.001 | < 0.001  (77.2, 8) |
|  | Non-mitochondrial proteins | 6026 | 4.52 | 4.761 | 2.40 |  |  |
| Hmax | Exclusive mitochondrial proteins | 492 | 4.650 | 4.539 | 1.58 | **0.007** | 0.022  (14.8, 6) |
|  | dual localized mitochondrial proteins | 181 | 4.360 | 4.143 | 1.82 |  |  |
|  | Mitochondrial proteins | 673 | 4.58 | 4.432 | 1.66 | 0.0016 | 0.0002  (26.06, 6) |
|  | Non-mitochondrial proteins | 6026 | 3.71 | 3.656 | 2.20 |  |  |
| Number of positively charged residues in N-terminus | Exclusive mitochondrial proteins | 492 | 6.00 | 6.51 | 4.17 | **< 0.001** | < 0.001  (46.2, 13) |
|  | dual localized mitochondrial proteins | 181 | 1.00 | 5.11 | 4.50 |  |  |
|  | Mitochondrial proteins | 673 | 5.00 | 5.923 | 3.73 | < 0.001 | < 0.001  (127.5, 13) |
|  | Non-mitochondrial proteins | 6026 | 0.00 | 2.455 | 3.17 |  |  |

The hydrophobic moment (μHδ), maxmimal hydrophobicity (Hmax), and the number of positive charged residues in the N-terminus are parameters used to evaluate the strength of mitochondrial targeting sequences (MTS, see text). Statistical analysis of differences between parameters of dual and exclusive mitochondrial proteins was carried out as in Supplementary Table 1S.
