# Supplementary material for: Dual Targeted Mitochondrial Proteins Are Characterized by Lower MTS Parameters and Total Net Charge
Source: PLoS One. 2008 May 14;3(5):e2161. doi: 10.1371/journal.pone.0002161 (PMC2367453; doi:10.1371/journal.pone.0002161)
Supplement: Table S3 — Dual targeted mitochondrial proteins have a lower total protein net charge than exclusive mitochondrial proteins. Statistical analysis of differences between parameters of dual and exclusive mitochondrial proteins was carried out as in Supplementary Table S1.* Differences are considered significant if p-value <0.05. (0.03 MB DOC) [file pone.0002161.s003.doc]

Table S3: Comparison of the total net charge of dual localized versus exclusive mitochondrial proteins in predicted mitochondrial proteins.

| Predicted protein location | Total Net Charge | | | | | |
| --- | --- | --- | --- | --- | --- | --- |
|  | N | Median | Mean | SD | p-value* (Mann-Whitney) | p-value*  (χ2 score, df) |
| Exclusive mitochondrial proteins | 492 | 6.00 | 6.94 | 4.17 | **< 0.001** | < 0.001  (43.9, 4) |
| dual localized mitochondrial proteins | 181 | 1.00 | 1.52 | 13.49 |  |  |
| Mitochondrial proteins | 673 | 5.00 | 5.49 | 11.49 | 0.0019 | 0.04  (10, 4) |
| Non-mitochondrial proteins | 6026 | 0 | -2.59 | 16.18 |  |  |

Dual targeted mitochondrial proteins have a lower total protein net charge than exclusive mitochondrial proteins. Statistical analysis of differences between parameters of dual and exclusive mitochondrial proteins was carried out as in Supplementary Table 1S.* Differences are considered significant if p-value < 0.05.
