# Supplementary material for: Dual Targeted Mitochondrial Proteins Are Characterized by Lower MTS Parameters and Total Net Charge
Source: PLoS One. 2008 May 14;3(5):e2161. doi: 10.1371/journal.pone.0002161 (PMC2367453; doi:10.1371/journal.pone.0002161)
Supplement: Table S4 — (0.25 MB DOC) [file pone.0002161.s004.doc]

Table S4: The reference set of dual-localized proteins

| **Name** | **Localization** | | **Motif** | **No. of Translation Products** | **2nd Met position** | **MuHd** | **Hmax** | **No. of basic residues** | **Net-Charge** | **MitoProtII score** | **Protein size** | **Proposed  targeting mechanism** | **Reference** |
| --- | --- | --- | --- | --- | --- | --- | --- | --- | --- | --- | --- | --- | --- |
|  | **Single Gene Studies** | **Prediction^a^** |  |  |  |  |  |  |  |  |  |  |  |
| ACO1 | M-C | M | MTS | 1 | 100 | 10.646 | 6.53 | 5 | 3 | 0.995 | 778 | Retrograde ? | (23) |
| ACS1 | M-C-P | M-N-C |  | 2 | 24 | 2.943 | 1.86 | 1 | -9 | 0.0206 | 713 | Translation initiation | (31) |
| ADK1 | M(IMS)-C | M-N-C | Weak-MTS | 1 | 8 | 5.634 | 1.77 | 2 | -3 | 0.0478 | 222 | Folding blocks entry | (26-28,32) |
| ALA1 | M-C | M-C | MTS | 2 | C - 49  M - 25 | C - 4.526  M - 9.403 | C - 3.9  M - 5.35 | C - 3  M - 7 | -25 | C - 0.1236  M - 0.8317 | 958 | Transcription initiation  Translation initiation – mitochondrial isoform initiated at two upstream in-frame ACG codons and provides function in both compartments | (33,34) |
| APN1 | M-N | M-N-C | NLS, Unknown MTS | 1 | 20 | 8.56 | 6.11 | 6 | 3 | 0.9685 | 367 | Pir1 binds NLS | (35,36) |
| CAT2 | M-P | M-P | MTS, PTS1 + Internal PTS | 2 | 22 | 7.398 | 3.83 | 6 | 4 | 0.8686 | 670 | Transcription initiation | (37-40) |
| CCA1 | M-C-N | M-C | MTS, NLS? | 2 | 9 | 4.53 | 3.87 | 4 | 3 | 0.956 | 546 | Transcription initiation | (41-43) |
| CDC9 | M-N | M-N-C | MTS, NLS | 2 | 23 | 7.611 | 4.17 | 14 | 1 | 0.963 | 755 | Transcription / translation  initiation | (44) |
| CCS1 | M(IMS)-C | N-C |  |  | 14 | 2.103 | 2.11 | 0 | -2 | 0.0119 | 249 | Mechanism unknown | (45,46) |
| COX17 | M(IMS)-C | M-N-C | No MTS  Cysteins signature | 1 | 57 | 1.859 | -1.94 | 0 | -5 | 0.001 | 69 | Trapped by disulfide relay system | (47) |
| COX19 | M(IMS)-C | M-N-C | No MTS  Cysteins signature | 1 | 33 | 2.229 | 2.85 | 1 | -4 | 0.0242 | 98 | Trapped by disulfide relay system | (48) |
| FUM1 | M-C | M-C | MTS | 1 | 23 | 8.385 | 4.56 | 7 | 4 | 0.926 | 488 | Retrograde | (49,50) |
| GLR1 | M-C | M-N-C | MTS | 2 | 16 | 7.49 | 3.8 | 10 | 1 | 0.501 | 483 | Translation initiation | (51) |
| GRS1 | M-C | M-C | MTS | 2 | C-70  M-23 | C-3.05  M-9.85 | C-1.88  M-3.54 | 0 | C-(-14)  M-(-7) | C-0.01  M-0.996 | 667 | Translation initiation | (52) |
| HTS1 | M-C | M-C | MTS | 2 | 20 | 7.731 | 5.44 | 12 | 1 | 0.988 | 546 | Transcription initiation | (53,54) |
| LAP3 | M-C | M-C | MTS |  | 146 | 2.895 | 2.95 | 0 | 1 | 0.101 | 454 | Mechanism unknown | (8,55-57) |
| LEU4 | M-C | M-C | MTS | 2 | 30 | 3.91 | 3.96 | 1 | -11 | 0.089 | 619 | Transcription initiation | (10,58-60) |
| MET7 | M-C | C | No predicted MTS |  | 16 | 8.952 | 7.86 | 6 | 8 | 0.312 | 548 | Mechanism unknown | (61,62) |
| MOD5 | M-C | M-N-C | MTS | 2 | 11 | 6.754 | 5.91 | 6 | 12 | 0.778 | 428 | Translation initiation | (63-66) |
| NAT2 | M-C | M-C | Predicted MTS |  | 1, 44 | 9.615 | 4.27 | 10 | 20 | 0.983 | 288 | Mechanism unknown | (8) |
| NFS1 | M-N | M | MTS, NLS |  | 84 | 7.811 | 4.42 | 7 | 3 | 0.986 | 497 | Mechanism unknown | (24,25,67) |
| NTG1 | M-N | M-N-C | Predicted MTS,  Unknown cleavage |  | 9 | 1.958 | 3.94 | 6 | 2 | 0.75 | 399 | Mechanism unknown | (68-70) |
| SOD1 | M(IMS)- P-N | N-C |  | 1 | 84 | 0.257 | 4.65 | 1 | -5 | 0.017 | 154 | Post-translational modifications  (i.e. zinc addition, copper insertion  and disulfide oxidation by CCS)  blocks import or leakage from IMS | (45,46,71-73) |
| TTR1 | M-C-ER | M-N | Predicted MTS |  | 34 | 1.135 | 4.96 | 0 | 0 | 0.01 | 570 | Mechanism unknown | (74) |
| VAS1 | M-C | M-C | MTS | 2 | 46 | 9.321 | 4.97 | 9 | 17 | 0.979 | 143 | Transcription initiation | (75) |
| RIB3 | M(IMS)-C | N-C | No simple MTS | 1 | 20 | 4.505 | 4.96 | 0 | -7 | 0.041 | 1104 | 2 functions 1) cytosolic riboflavin biosynthesis. 2)mitochondrial respiration. Both products looks the same on PAGE. Mechanism unknown | (76) |
| YHB1 | M-C | C | No MTS |  | 31 | 8.128 | 5.5 | 5 | -10 | 0.169 | 399 | Mechanism unknown | (77) |
| YNK1 | M(IMS)-C | N-C | No classical MTS | 1 | 64 | 4.314 | 3.14 | 0 | 2 | 0.1 | 153 | Phosphorilation inhibits mitochondrial import by lowering affinity for TOM40 | (78) |
| TRM1 | M-N | N | NLS,  Uncleaved MTS,  functional w/o first 16 aa | 2 | 16 | 6.42 | 5.545 | 7 | 18 | 0.572 | 778 | Both products  targeted to mitochondria,  AUG16 less efficiently.  Distribution mechanism  unknown | (79-83) |

**References**

1. Danpure, C. J. (1995) *Trends Cell Biol* **5**(6), 230-238

2. Small, I., Wintz, H., Akashi, K., and Mireau, H. (1998) *Plant Mol Biol* **38**(1-2), 265-277

3. Kragler, F., Langeder, A., Raupachova, J., Binder, M., and Hartig, A. (1993) *J Cell Biol* **120**(3), 665-673

4. Petrova, V. Y., Drescher, D., Kujumdzieva, A. V., and Schmitt, M. J. (2004) *Biochem J* **380**(Pt 2), 393-400

5. Karniely, S., and Pines, O. (2005) *EMBO Rep* **6**(5), 420-425

6. Pfanner, N., and Geissler, A. (2001) *Nat Rev Mol Cell Biol* **2**(5), 339-349

7. Prokisch, H., Scharfe, C., Camp, D. G., 2nd, Xiao, W., David, L., Andreoli, C., Monroe, M. E., Moore, R. J., Gritsenko, M. A., Kozany, C., Hixson, K. K., Mottaz, H. M., Zischka, H., Ueffing, M., Herman, Z. S., Davis, R. W., Meitinger, T., Oefner, P. J., Smith, R. D., and Steinmetz, L. M. (2004) *PLoS Biol* **2**(6), e160

8. Sickmann, A., Reinders, J., Wagner, Y., Joppich, C., Zahedi, R., Meyer, H. E., Schonfisch, B., Perschil, I., Chacinska, A., Guiard, B., Rehling, P., Pfanner, N., and Meisinger, C. (2003) *Proc Natl Acad Sci U S A* **100**(23), 13207-13212

9. Huh, W. K., Falvo, J. V., Gerke, L. C., Carroll, A. S., Howson, R. W., Weissman, J. S., and O'Shea, E. K. (2003) *Nature* **425**(6959), 686-691

10. Kumar, A., Agarwal, S., Heyman, J. A., Matson, S., Heidtman, M., Piccirillo, S., Umansky, L., Drawid, A., Jansen, R., Liu, Y., Cheung, K. H., Miller, P., Gerstein, M., Roeder, G. S., and Snyder, M. (2002) *Genes Dev* **16**(6), 707-719

11. Abbas-Terki, T., and Picard, D. (1999) *Eur J Biochem* **266**(2), 517-523

12. Karniely, S., Rayzner, A., Sass, E., and Pines, O. (2006) *Exp Cell Res* **312**(19), 3835-3846

13. Daum, G., Bohni, P. C., and Schatz, G. (1982) *J Biol Chem* **257**(21), 13028-13033

14. Andreoli, C., Prokisch, H., Hortnagel, K., Mueller, J. C., Munsterkotter, M., Scharfe, C., and Meitinger, T. (2004) *Nucleic Acids Res* **32**(Database issue), D459-462

15. Prokisch, H., Andreoli, C., Ahting, U., Heiss, K., Ruepp, A., Scharfe, C., and Meitinger, T. (2006) *Nucleic Acids Res* **34**(Database issue), D705-711

16. Claros, M. G., and Vincens, P. (1996) *Eur J Biochem* **241**(3), 779-786

17. Neupert, W. (1997) *Annu Rev Biochem* **66**, 863-917

18. Roise, D., Theiler, F., Horvath, S. J., Tomich, J. M., Richards, J. H., Allison, D. S., and Schatz, G. (1988) *Embo J* **7**(3), 649-653

19. von Heijne, G. (1986) *Embo J* **5**(6), 1335-1342

20. Hartmann, C., Christen, P., and Jaussi, R. (1991) *Nature* **352**(6338), 762-763

21. Jaussi, R. (1995) *Eur J Biochem* **228**(3), 551-561

22. Regev-Rudzki, N., and Pines, O. (2007) *Bioessays* **29**(8), 772-782

23. Regev-Rudzki, N., Karniely, S., Ben-Haim, N. N., and Pines, O. (2005) *Mol Biol Cell* **16**(9), 4163-4171

24. Muhlenhoff, U., Balk, J., Richhardt, N., Kaiser, J. T., Sipos, K., Kispal, G., and Lill, R. (2004) *J Biol Chem* **279**(35), 36906-36915

25. Nakai, Y., Nakai, M., Hayashi, H., and Kagamiyama, H. (2001) *J Biol Chem* **276**(11), 8314-8320

26. Bandlow, W., Strobel, G., and Schricker, R. (1998) *Biochem J* **329 (Pt 2)**, 359-367

27. Magdolen, V., Schricker, R., Strobel, G., Germaier, H., and Bandlow, W. (1992) *FEBS Lett* **299**(3), 267-272

28. Schricker, R., Angermayr, M., Strobel, G., Klinke, S., Korber, D., and Bandlow, W. (2002) *J Biol Chem* **277**(32), 28757-28764

29. Cabantous, S., Terwilliger, T. C., and Waldo, G. S. (2005) *Nat Biotechnol* **23**(1), 102-107

30. Zhang, S., Ma, C., and Chalfie, M. (2004) *Cell* **119**(1), 137-144

31. De Virgilio, C., Burckert, N., Barth, G., Neuhaus, J. M., Boller, T., and Wiemken, A. (1992) *Yeast* **8**(12), 1043-1051

32. Cooper, A. J., and Friedberg, E. C. (1992) *Gene* **114**(1), 145-148

33. Chang, K. J., Lin, G., Men, L. C., and Wang, C. C. (2006) *J Biol Chem* **281**(12), 7775-7783

34. Huang, H. Y., Tang, H. L., Chao, H. Y., Yeh, L. S., and Wang, C. C. (2006) *Mol Microbiol* **60**(1), 189-198

35. Ramotar, D., Kim, C., Lillis, R., and Demple, B. (1993) *J Biol Chem* **268**(27), 20533-20539

36. Vongsamphanh, R., Fortier, P. K., and Ramotar, D. (2001) *Mol Cell Biol* **21**(5), 1647-1655

37. Atomi, H., Ueda, M., Suzuki, J., Kamada, Y., and Tanaka, A. (1993) *FEMS Microbiol Lett* **112**(1), 31-34

38. Elgersma, Y., van Roermund, C. W., Wanders, R. J., and Tabak, H. F. (1995) *Embo J* **14**(14), 3472-3479

39. Kispal, G., Sumegi, B., Dietmeier, K., Bock, I., Gajdos, G., Tomcsanyi, T., and Sandor, A. (1993) *J Biol Chem* **268**(3), 1824-1829

40. Schafer, H., Nau, K., Sickmann, A., Erdmann, R., and Meyer, H. E. (2001) *Electrophoresis* **22**(14), 2955-2968

41. Chen, J. Y., Joyce, P. B., Wolfe, C. L., Steffen, M. C., and Martin, N. C. (1992) *J Biol Chem* **267**(21), 14879-14883

42. Wolfe, C. L., Hopper, A. K., and Martin, N. C. (1996) *J Biol Chem* **271**(9), 4679-4686

43. Wolfe, C. L., Lou, Y. C., Hopper, A. K., and Martin, N. C. (1994) *J Biol Chem* **269**(18), 13361-13366

44. Willer, M., Rainey, M., Pullen, T., and Stirling, C. J. (1999) *Curr Biol* **9**(19), 1085-1094

45. Field, L. S., Furukawa, Y., O'Halloran, T. V., and Culotta, V. C. (2003) *J Biol Chem* **278**(30), 28052-28059

46. Sturtz, L. A., Diekert, K., Jensen, L. T., Lill, R., and Culotta, V. C. (2001) *J Biol Chem* **276**(41), 38084-38089

47. Beers, J., Glerum, D. M., and Tzagoloff, A. (1997) *J Biol Chem* **272**(52), 33191-33196

48. Nobrega, M. P., Bandeira, S. C., Beers, J., and Tzagoloff, A. (2002) *J Biol Chem* **277**(43), 40206-40211

49. Sass, E., Blachinsky, E., Karniely, S., and Pines, O. (2001) *J Biol Chem* **276**(49), 46111-46117

50. Sass, E., Karniely, S., and Pines, O. (2003) *J Biol Chem* **278**(46), 45109-45116

51. Outten, C. E., and Culotta, V. C. (2004) *J Biol Chem* **279**(9), 7785-7791

52. Chang, K. J., and Wang, C. C. (2004) *J Biol Chem* **279**(14), 13778-13785

53. Chiu, M. I., Mason, T. L., and Fink, G. R. (1992) *Genetics* **132**(4), 987-1001

54. Natsoulis, G., Hilger, F., and Fink, G. R. (1986) *Cell* **46**(2), 235-243

55. Enenkel, C., and Wolf, D. H. (1993) *J Biol Chem* **268**(10), 7036-7043

56. Niemer, I., Muller, G., Strobel, G., and Bandlow, W. (1997) *Curr Genet* **32**(1), 41-51

57. Zheng, W., and Johnston, S. A. (1998) *Mol Cell Biol* **18**(6), 3580-3585

58. Beltzer, J. P., Chang, L. F., Hinkkanen, A. E., and Kohlhaw, G. B. (1986) *J Biol Chem* **261**(11), 5160-5167

59. Beltzer, J. P., Morris, S. R., and Kohlhaw, G. B. (1988) *J Biol Chem* **263**(1), 368-374

60. dos Santos, M. M., Gombert, A. K., Christensen, B., Olsson, L., and Nielsen, J. (2003) *Eukaryot Cell* **2**(3), 599-608

61. Cherest, H., Thomas, D., and Surdin-Kerjan, Y. (2000) *J Biol Chem* **275**(19), 14056-14063

62. DeSouza, L., Shen, Y., and Bognar, A. L. (2000) *Arch Biochem Biophys* **376**(2), 299-312

63. Gillman, E. C., Slusher, L. B., Martin, N. C., and Hopper, A. K. (1991) *Mol Cell Biol* **11**(5), 2382-2390

64. Najarian, D., Dihanich, M. E., Martin, N. C., and Hopper, A. K. (1987) *Mol Cell Biol* **7**(1), 185-191

65. Slusher, L. B., Gillman, E. C., Martin, N. C., and Hopper, A. K. (1991) *Proc Natl Acad Sci U S A* **88**(21), 9789-9793

66. Zoladek, T., Vaduva, G., Hunter, L. A., Boguta, M., Go, B. D., Martin, N. C., and Hopper, A. K. (1995) *Mol Cell Biol* **15**(12), 6884-6894

67. Nakai, Y., Umeda, N., Suzuki, T., Nakai, M., Hayashi, H., Watanabe, K., and Kagamiyama, H. (2004) *J Biol Chem* **279**(13), 12363-12368

68. Alseth, I., Eide, L., Pirovano, M., Rognes, T., Seeberg, E., and Bjoras, M. (1999) *Mol Cell Biol* **19**(5), 3779-3787

69. Meadows, K. L., Song, B., and Doetsch, P. W. (2003) *Nucleic Acids Res* **31**(19), 5560-5567

70. You, H. J., Swanson, R. L., Harrington, C., Corbett, A. H., Jinks-Robertson, S., Senturker, S., Wallace, S. S., Boiteux, S., Dizdaroglu, M., and Doetsch, P. W. (1999) *Biochemistry* **38**(35), 11298-11306

71. Culotta, V. C., Klomp, L. W., Strain, J., Casareno, R. L., Krems, B., and Gitlin, J. D. (1997) *J Biol Chem* **272**(38), 23469-23472

72. Nedeva, T. S., Petrova, V. Y., Zamfirova, D. R., Stephanova, E. V., and Kujumdzieva, A. V. (2004) *FEMS Microbiol Lett* **230**(1), 19-25

73. O'Brien, K. M., Dirmeier, R., Engle, M., and Poyton, R. O. (2004) *J Biol Chem* **279**(50), 51817-51827

74. Pedrajas, J. R., Porras, P., Martinez-Galisteo, E., Padilla, C. A., Miranda-Vizuete, A., and Barcena, J. A. (2002) *Biochem J* **364**(Pt 3), 617-623

75. Chatton, B., Walter, P., Ebel, J. P., Lacroute, F., and Fasiolo, F. (1988) *J Biol Chem* **263**(1), 52-57

76. Jin, C., Barrientos, A., and Tzagoloff, A. (2003) *J Biol Chem* **278**(17), 14698-14703

77. Cassanova, N., O'Brien, K. M., Stahl, B. T., McClure, T., and Poyton, R. O. (2005) *J Biol Chem* **280**(9), 7645-7653

78. Amutha, B., and Pain, D. (2003) *Biochem J* **370**(Pt 3), 805-815

79. Ellis, S. R., Hopper, A. K., and Martin, N. C. (1989) *Mol Cell Biol* **9**(4), 1611-1620

80. Li, J. M., Hopper, A. K., and Martin, N. C. (1989) *J Cell Biol* **109**(4 Pt 1), 1411-1419

81. Rose, A. M., Belford, H. G., Shen, W. C., Greer, C. L., Hopper, A. K., and Martin, N. C. (1995) *Biochimie* **77**(1-2), 45-53

82. Rose, A. M., Joyce, P. B., Hopper, A. K., and Martin, N. C. (1992) *Mol Cell Biol* **12**(12), 5652-5658

83. Stanford, D. R., Martin, N. C., and Hopper, A. K. (2000) *Nucleic Acids Res* **28**(2), 383-392
