# Supplementary material for: Dual Targeted Mitochondrial Proteins Are Characterized by Lower MTS Parameters and Total Net Charge
Source: PLoS One. 2008 May 14;3(5):e2161. doi: 10.1371/journal.pone.0002161 (PMC2367453; doi:10.1371/journal.pone.0002161)
Supplement: Table S5 — (0.05 MB DOC) [file pone.0002161.s005.doc]

Table S5A: Characteristics of 9 proteins comprising a subgroup of predicted dual localized proteins

| **Protein** | **Function*** | **MitoProtII score** | **μHδ** | **Hmax** | **No. of basic residues** | **NET-CHARGE** | **2^nd^ Met position** | **Prokaryote homology starting point** |
| --- | --- | --- | --- | --- | --- | --- | --- | --- |
| PRD1 | Zinc metallo-endopeptidase | 0.785 | 4.88 | 4.09 | 3 | -18 | 26 | 40 |
| HNT2 | Dinucleoside triphosphate hydrolase | 0.796 | 4.13 | 3.33 | 10 | 4 | 11 | 29 |
| MGE1 | Mitochondrial GrpE | 0.998 | 5.73 | 3.85 | 9 | 1 | 19 | 62 |
| FMP40 | Unknown | 0.891 | 4.65 | 3.26 | 6 | -19 | 57 | 64 |
| GLO4 | Glyoxalase | 0.766 | 7.35 | 5.36 | 5 | 0 | 10 | 26 |
| GPD2 | NAD-dependent glycerol 3-phosphate dehyrogenase | 0.993 | 9.14 | 2.74 | 14 | -4 | 48 | 86 |
| LPD1 | Dihydrolipoamide dehydrogenase | 0.945 | 9.88 | 4.22 | 11 | 2 | 85 | 26 |
| AEP1 | Protein required for expression of Oli1 gene encoding subunit 9 of F1-F0 ATP synthase | 0.975 | 7.99 | 4.71 | 7 | 18 | 18 | 124 |
| YGR031W | Unknown | 0.787 | 9.20 | 6.44 | 6 | 12 | 1 | 70 |

Table S5B: Characteristics of 5 proteins comprising a subgroup of predicted exclusive mitochondrial proteins

| **Protein** | **Function** | **MitoProtII score** | **μHδ** | **Hmax** | **No. of basic residues** | **NET-CHARGE** | **2^nd^ Met position** | **Prokaryote homology starting point** |
| --- | --- | --- | --- | --- | --- | --- | --- | --- |
| FMP36 | Unknown | 0.916 | 11.01 | 4.71 | 6 | 9 | 35 | 0 |
| MSS116 | Splicing of mitochondrial Group I and II introns | 0.857 | 6.86 | 5.56 | 5 | 13 | 123 | 102 |
| ACN9 | Required for acetate utilization and gluconeogenesis | 0.989 | 9.15 | 5.09 | 8 | 3 | 43 | 35 |
| MRPL11 | Mitochondrial ribosomal protein of the large subunit | 0.529 | 7.64 | 5.35 | 5 | 13 | 6 | 49 |
| GCV3 | H subunit of the mitochondrial glycine decarboxylase complex. | 0.998 | 7.14 | 3.47 | 8 | -11 | 11 | 47 |
